# Support service utilization and out-of-pocket payments for health services in a population-based sample of adults with neurological conditions

**DOI:** 10.1371/journal.pone.0192911

**Published:** 2018-02-23

**Authors:** Adebimpe O. Obembe, Charlie H. Goldsmith, Lisa A. Simpson, Brodie M. Sakakibara, Janice J. Eng

**Affiliations:** 1 Department of Physical Therapy, The University of British Columbia, Vancouver, Canada; 2 Rehabilitation Research Program, GF Strong Rehab Centre, Vancouver Coastal Health Research Institute, Vancouver, Canada; 3 Adjunct Professor, Faculty of Health Sciences, Simon Fraser University, Burnaby, Canada; 4 Adjunct Professor, Department of Occupational Science and Occupational Therapy, The University of British Columbia, Vancouver, Canada; 5 Graduate Program in Rehabilitation Sciences, The University of British Columbia, Vancouver, Canada; Harvard University, UNITED STATES

## Abstract

**Background:**

Social support can help to deal with the consequences of neurological conditions and promote functional independence and quality of life. Our aim was to evaluate the impact of neurological conditions on the use of support and health-care services in a population-based sample of community-dwelling adults with neurological conditions.

**Methods:**

Data were from the Survey of Living with Neurological Conditions in Canada, which was derived from a representative sample of household residents. Formal and informal support received and out-of-pocket payments were assessed by personal interviews. Logistic regression was used to explore the association between support service utilization and six common neurological conditions (Stroke, Parkinson's disease, Alzheimer's disease/dementias, traumatic brain injury, spinal cord injury and multiple sclerosis) with stroke as the reference category.

**Results:**

The sample contained 2,410 respondents and equate to an estimated 459,770 when sample weights were used. A larger proportion of people within each of the neurological conditions received informal support than formal support (at least twice as much). Samples with the non-stroke conditions were more likely to receive formal assistance for personal (odds ratios 2.7 to 5.6; P < 0.05) and medical (odds ratios 2.4 to 4.4; P < 0.05) care compared to the stroke group. Also, the non-stroke conditions were more likely to receive informal assistance (odds ratios 2.7 to 17.9; P < 0.05) and less likely to make out-of-pocket payments for rehabilitation therapy (odds ratios 0.2 to 0.3; P < 0.05) than the stroke group. The Alzheimer’s disease/dementia group had the highest proportion who received formal and informal support services.

**Conclusions:**

Our findings suggest that Canadians with neurological conditions receive more informal assistance than formal assistance. Furthermore, it appears that stroke survivors receive less support services, while those with Alzheimer’s disease/dementia receive the most compared to other adult neurological conditions. Such data can help inform the development of support services in the community.

## Introduction

An estimated 3.6 million Canadians living in the community are affected by neurological conditions [1]. Many neurological disorders and conditions result in long-term impairments that limit functional abilities and restrict participation [2]. Assistance and support services are necessities for participating in society for many people with disabilities [3]. Many of these individuals need assistance and support to achieve a good quality of life and to be able to participate in social and economic life on an equal basis with others [4]. Yet despite this, it is widely recognized that health-care systems lack continuity across services and are often criticized for shortening hospital length-of-stay and offering limited community services [5]. Moreover, these individuals may be faced with additional health-related financial challenges [6] (e.g. expenses for assistive technology and home care services) after returning home if they lack full independence. The lack of necessary support services can make people with disabilities excessively dependent on family members, which can prevent both the person with disability and the family members from becoming economically and socially involved [3]. The prevalence and incidence of some of the most common neurological conditions tend to increase with age [1]. Consequently, the demand on Canadian families, the healthcare system and the economy will increase [7] as both the number of individuals facing the challenges associated with neurological conditions and the cost of caring for these individuals are expected to rise [1].

Neurological conditions can be devastating to individuals and their family members depriving them of their independence. The cost of healthcare in Canada is rising, and the impact on Canadians with neurological conditions is not known. A better understanding of the utilization of support services is essential for improving access to care and reducing financial burden on patients and their families. Therefore, the purpose of this study was to describe the amount of support services received by community-dwelling individuals with neurological conditions, as well as their out-of-pocket healthcare payments. We compared the utilization of a) formal support services, b) informal support services and c) out-of-pocket health service payments among six adult neurological conditions.

## Methods

### Data source

Data were from the 2011/2012 Survey of Living with Neurological Conditions in Canada (SLNCC). Secondary analysis, using Statistics Canada data, did not require an ethics review, however, a proposal on the use of the data was approved by Statistics Canada. The SLNCC is a nationally representative, population-based survey designed to collect information about experiences with 18 chronic neurological conditions, among Canadians aged 15 years or older living in private households, using computer-assisted telephone interviews. Participants with neurological conditions were identified from the 2010/2011 Canadian Community Health Survey (CCHS) and were recruited to participate in the SLNCC. The SLNCC is a nationally representative, population-based survey of ~5,100 Canadians with at least one neurological condition, and had a response rate of 81.6%. Respondents were asked whether they had one of 18 neurological conditions diagnosed by a health professional that had lasted or was expected to last at least six months [8]. The information was collected directly from the respondents. Proxy interviews were permitted for the SLNCC. Detailed descriptions of the survey are available elsewhere [8,9].

### Inclusion and exclusion criteria

Our inclusion criteria required that individuals be at least 18 years of age at the time of the interview, and have exactly one of the common neurological conditions that lead to impaired physical and/or cognitive function in Canada [1]: stroke, Parkinson's disease (PD), Alzheimer's disease and other dementias (ADD), traumatic brain injury (TBI), spinal cord injury (SCI) or multiple sclerosis (MS). We excluded people with more than one of the five conditions because it would be difficult to separate the effects from other conditions. We did not exclude individuals who had migraines in addition to these conditions as migraines commonly present with neurological conditions [10]. Individuals were excluded if the neurological conditions were congenital or primarily diagnosed during childhood, such as muscular dystrophy and epilepsy.

### Socio-demographic variables

Respondents were asked to provide information on age, sex, educational status, income (total household income) and activity restrictions. Respondents were asked if they experienced restrictions in at least one usual activity (such as driving, educational and job opportunities) caused by their neurological condition.

### Support service and health-care utilization measures

Respondents were asked if they received formal (from paid workers or employees, or volunteer organizations) and informal (from family, friends, or neighbors) assistance (yes/no) in the past 12 months at home, work or school because of their neurological condition [8,9]. The following six categories of assistance were queried for both formal assistance and informal assistance: 1) personal care (such as assistance with eating, dressing, or bathing), 2) medical care (such as help taking medicine or help with nursing care), 3) managing care (such as making appointments or managing personal finances), 4) transportation (including trips to the doctor or shopping), 5) emotional support and 6) household activities (such as housework, meal preparation, or outdoor work).

Respondents were asked to indicate if they had made any out-of-pocket payment (yes/no) related to their condition in the past 12 months in the following four areas: 1) medication, 2) assistive devices, 3) rehabilitation therapy and 4) home care services.

### Statistical analysis

Descriptive statistics were used to summarize the data. A two-part statistical process was then undertaken: Part 1) determining covariates that may interact with the logistic regression and Part 2) using logistic regression controlling for covariates to determine differences between the six neurological conditions for utilization of a) formal support services, b) informal support services and c) out-of-pocket payments for health services.

Part 1: The covariates were selected by a two-step process. We initially selected the covariates based on the literature, e.g., older individuals, women, and those with lower incomes have poorer functioning [11] and quality of life [12]. We selected the final covariates by using smoother curves (LOESS—locally weighted scatter-plot smoothing) [13]. LOESS, a descriptive, exploratory tool for fitting smooth curves to scatter plots, was used to provide a graphical summary of the relationship between the dependent variable (each of formal and informal assistance, and out-of-pocket payments for health services) and covariates (age, income, education and activity restrictions). The LOESS curves showed a negative relationship between assistance received, and age and income. Assistance received approached the minimum value at about 60 years for age and $40,000 for income, and thereafter gradually increased. Based on the results of the LOESS smoothed curves, education and activity restrictions were excluded, while age (< 60 years and ≥ 60 years) and income (< $40 000 and ≥ $40 000) were grouped into two categories and were retained as covariates, including sex (male and female).

Part 2: Logistic regression models with adjusted odds ratios (OR) and 95% confidence intervals (CI) were created to investigate the relationship between dependent variables (each of formal and informal assistance, and out-of-pocket payments for health services) and the independent variable (6 levels of the selected neurological conditions, with stroke as the reference category). Stroke was selected as the reference category as it represented the largest cohort in the sample. Age, sex and income were entered into the model to control for socio-demographic covariates prior to the independent variable.

Multiple imputation method was used to model missing values using all the variables (independent and dependent) as predictors [14], with fully conditional specification.

Replicate sampling weights developed for the SLNCC by Statistics Canada and bootstrapped variance estimation were used to ensure that the results were representative of people with neurological conditions in Canada, and to account for survey design effects. Confidence intervals were presented for the multiple comparisons, as the sampling error would be less critical in this weighted representative population-based sample. Alpha level was set at 0.05. All analyses were performed with the IBM Statistical Package for Social Sciences (SPSS) for Windows, Version 22.0 and WesVar 5.1 software package.

## Results

### Sample characteristics

Sample characteristics are presented in Table 1. 8.7% of respondents with the selected neurological conditions were excluded because they had more than one of the six conditions. The stroke (38%) and ADD (37.8%) groups had the most proxy respondents, while the SCI (2%) and MS (3.3%) groups had the least proxy respondents. Analysis of the models with multiple imputation showed similar results with complete-case analysis for the final models produced. The final sample consisted of 2,410 respondents and equate to an estimated 459,770 when sampling weights were used. The sample had a mean (SD) age of 61.4 (17.2) years and 52.9% were women. Stroke was the most prevalent condition in the sample (38.4%) while PD was the least prevalent condition (8.9%). The majority of stroke survivors (86.1%) were restricted in activities, and this proportion was higher than the PD, MS and ADD groups, but lower than those with neurotrauma (TBI and SCI).

**Table 1:** Characteristics of samples from the survey of living with neurological conditions in Canada.

N (%)*	Overall 459770 (100)	Stroke 176452 (38.4)	Parkinson's disease 40753 (8.9)	Traumatic Brain Injury 61929 (13.5)	Spinal Cord Injury 50967(11.1)	Multiple sclerosis 83957 (18.3)	Alzheimer's and dementias 45712 (9.9)
**Age (years)**							
Mean (SD)	61.4 (17.2)	67.0 (13.8)	72.8 (9.7)	44.2 (16.9)	53.2 (14.2)	52.0 (12.4)	79.5 (7.6)
< 60 (%)	42.8	25.2	8.1	82.7	70.4	72.6	2.1
≥ 60 (%)	57.2	74.8	91.9	17.3	29.6	27.4	97.9
**Sex**							
Male (%)	47.1	47.3	64.5	52.5	66.1	26.7	39.5
Female (%)	52.9	52.7	35.5	47.5	33.9	73.3	60.5
**Education**							
≤ High school (%)	23.8	27.6	27.5	19.5	16.9	12.0	41.5
> High school (%)	76.2	72.4	72.5	80.5	83.1	88.0	58.5
**Total household Income**^**†**^							
< $40000 (%)	40.4	44.8	30.2	45.7	37.0	37.1	34.6
≥ $40000 (%)	44.3	38.6	49.5	41.0	50.6	51.8	45.8
Missing (%)	15.3	16.6	20.2	13.3	12.4	11.1	19.6
**Restriction in activities**^**†**^							
Yes (%)	86.7	86.1	76.6	93.1	98.1	83.4	83.1
No (%)	12.7	13.5	21.9	6.7	1.9	15.5	16.3
Missing (%)	0.6	0.4	1.5	0.2	0.0	1.1	0.6

*Replicate sampling weights were used to develop a population-based distribution

^†^Some respondents did not provide responses to the question

Upper limb impairment was similar across groups, with 90.2% to 96.2% able to grasp and handle small objects. Thirty to forty percent of each group required mechanical support to walk (e.g., cane or walker), with the exception of those with traumatic brain injury, which was less (10%)

### Effect of neurological conditions on support services and out-of-pocket payments

#### Formal support service

The stroke group had the lowest proportion of people who received at least one category of formal assistance (17.5%), while the ADD group had the highest proportion (40%) (Table 2). In fact, the ADD group had the highest proportion who received formal support services in three of the six categories of assistance. For formal support services, upon first entering the covariates (age, sex and income) into the logistic regressions, younger age (≤ 60 years) and higher income were associated with higher odds of receiving assistance with personal and medical care S1 Table. After controlling for age, sex and income, the PD, SCI, and ADD groups were approximately three to six times more likely to receive formal assistance with personal care (OR 2.8–5.6; p < 0.05) and at least twice as likely to receive formal assistance with medical care (OR 2.4–4.4; p < 0.05) compared to the stroke group (Table 2).

**Table 2:** Formal support service use reported by samples in the survey of living with neurological conditions in Canada.

	Overall (N = 459770)	Stroke^§^ (N = 176452)	Parkinson’s disease (N = 40753)	Traumatic Brain Injury (N = 61929)	Spinal Cord Injury (N = 50967)	Multiple sclerosis (N = 83957)	Alzheimer's and dementias (N = 45712)
**Percent**^**†**^							
Personal care	11.0	8.9	20.5	4.8	8.2	10.7	25.0
Medical care	8.1	5.7	11.2	4.5	8.7	7.5	21.0
Managing care	4.1	1.4	4.8	13.1	2.0	3.9	4.7
Transportation	7.6	5.3	8.3	14.1	9.2	7.3	8.2
Emotional support	6.9	4.5	7.7	12.6	4.1	7.7	10.5
Household activities	13.3	10.1	16.9	13.1	15.2	15.3	17.0
At least one care service	22.6	17.5	28.6	21.6	25.3	0.0	40.0
**OR (95% CI)**^**‡**^ **[p-value]**							
Personal care			2.8 (1.5, 5.2) [0.001]	1.2 (0.6, 2.5) [0.576]	5.6 (1.7, 19.0) [0.006]	2.7 (1.1, 6.3) [0.027]	2.8 (1.3, 6.1) [0.009]
Medical care			3.6 (1.7, 7.6) [0.001]	1.8 (0.7, 4.5) [0.201]	4.4 (1.1, 17.9) [0.037]	1.9 (0.8, 4.6) [0.185]	2.4 (1.1, 5.2) [0.023]
Managing care			3.9 (0.9, 18.2) [0.080]	0.8 (0.2, 3.2) [0.746]	0.5 (0.1, 2.2) [0.383]	4.4 (0.8, 24.4) [0.091]	2.5 (0.6, 10.3) [0.191]
Transportation			1.7 (0.6, 4.7) [0.336]	1.3 (0.4, 4.7) [0.664]	0.6 (0.2, 2.3) [0.496]	1.1 (0.4, 3.0) [0.912]	1.4 (0.5, 3.5) [0.514]
Emotional support			2.1 (0.7, 6.4) [0.186]	1.2 (0.4, 3.2) [0.757]	0.8 (0.2, 2.5) [0.653]	2.2 (0.5, 10.3) [0.303]	2.1 (0.8, 5.5) [0.128]
Household activities			1.5 (0.7, 3.2) [0.281]	0.9 (0.4, 1.9) [0.789]	0.7 (0.3, 1.9) [0.515]	0.7 (0.3, 1.5) [0.386]	0.9 (0.5, 2.0) [0.894]

^†^Weighted percentages

^‡^Odds ratios were adjusted for age, sex and income

^§^Reference category

#### Informal support service

Generally, a larger proportion of people within each of the six neurological conditions received informal support than formal support (at least twice as much). The stroke group had the second lowest proportion of people who received at least one category of informal assistance (47.7%) with the ADD group having the highest proportion (82.4%) (Table 3). The ADD group had the highest proportion who received informal support services in all six categories. For informal support services, younger age was associated with higher proportions of people receiving assistance with personal, medical and managing care. In addition, female sex was associated with higher proportions of people receiving informal assistance with transportation and emotional support S2 Table. The five non-stroke neurological conditions were between two to eighteen times more likely to receive any type of informal assistance (OR 2.1 to 17.9; p < 0.05) except for personal care when compared to the stroke group. Each of the five non-stroke neurological conditions was particularly more likely to receive informal assistance for medical (OR 7.9 to 10.6; p < 0.05) and managing (OR 6.5 to 17.9; p < 0.05) care (Table 3).

**Table 3:** Informal support service use reported by samples in the survey of living with neurological conditions in Canada.

	Overall (N = 459770)	Stroke^§^ (N = 176452)	Parkinson's disease (N = 40753)	Traumatic Brain Injury (N = 61929)	Spinal Cord Injury (N = 50967)	Multiple sclerosis (N = 83957)	Alzheimer's and dementias (N = 45712)
**Percent**^**†**^							
Personal care	20.5	18.4	25.4	11.3	23.9	15.1	43.7
Medical care	20.8	17.9	20.8	15.2	13.6	10.4	67.6
Managing care	29.1	24.7	31.3	32.7	23.0	14.6	75.0
Transportation	40.0	34.6	35.9	39.3	40.3	33.6	77.4
Emotional support	42.6	38.0	42.5	39.2	43.1	37.1	74.2
Household activities	45.0	37.3	42.1	43.7	56.8	40.2	74.4
At least one care service	52.4	47.7	48.4	49.3	54.5	46.2	82.4
**OR (95% CI)**^**‡**^ **[p-value]**							
Personal care			2.7 (1.6, 4.6) [<0.001]	1.7 (0.9, 3.2) [0.081]	5.3 (2.3, 12.3) [<0.001]	1.8 (1.0, 3.4) [0.065]	3.0 (1.5, 6.3) [0.003]
Medical care			7.9 (4.5, 13.8) [<0.001]	7.9 (4.1, 15.4) [<0.001]	8.7 (3.6, 21.1) [<0.001]	9.8 (4.7, 20.4) [<0.001]	10.6 (5.0, 22.5) [<0.001]
Managing care			10.3 (5.8, 18.1) [<0.001]	7.4 (3.8, 14.3) [<0.001]	6.5 (2.8, 15.2) [<0.001]	9.7 (4.7, 19.9) [<0.001]	17.9 (8.9, 36.1) [<0.001]
Transportation			6.5 (3.5, 12.4) [<0.001]	3.9 (2.1, 7.4) [<0.001]	4.9 (2.3, 10.7) [<0.001]	3.7 (1.8, 7.6) [<0.001]	7.6 (3.9, 14.4) [<0.001]
Emotional support			4.4 (2.4, 7.9) [<0.001]	3.1 (1.6, 5.8) [0.001]	4.6 (2.1, 10.1) [<0.001]	3.3 (1.6, 6.6) [0.001]	5.1 (2.7, 9.9) [<0.001]
Household activities			4.9 (2.7, 9.1) [<0.001]	3.3 (1.8, 6.1) [<0.001]	4.0 (1.9, 8.6) [<0.001]	2.1 (1.0, 4.4) [0.039]	4.5 (2.4, 8.2) [<0.001]

^†^Weighted percentages

^‡^Odds ratios were adjusted for age, sex and income

^§^Reference category

#### Out-of-pocket payments

For out-of-pocket expenses, a lower income was associated with having more rehabilitation therapy expenses S3 Table. More than half of the stroke group (54.2%) made out-of-pocket payments in at least one of the four expense categories. People with the five non-stroke neurological conditions were approximately three to seven times less likely to make out-of-pocket payments for rehabilitation therapy (OR 0.2 to 0.3; P < 0.05) than the stroke group (Table 4).

**Table 4:** Out-of-pocket payments reported by samples in the survey of living with neurological conditions in Canada.

	Overall (N = 459770)	Stroke^§^ (N = 176452)	Parkinson's disease (N = 40753)	Traumatic Brain Injury (N = 61929)	Spinal Cord Injury (N = 50967)	Multiple sclerosis (N = 83957)	Alzheimer's and dementias (N = 45712)
**Percent**^**†**^							
Medication	43.9	43.2	44.3	33.6	52.6	44.2	50.2
Assistive devices	17.9	15.6	23.2	11.6	24.0	22.8	17.4
Rehabilitation therapy	15.8	14.7	17.3	15.6	24.5	19.0	5.3
Home care services	7.8	5.2	14.1	5.6	9.5	7.9	14.2
At least one category of payment	39.4	54.2	59.5	39.4	62.7	59.4	60.6
**OR (95% CI)**^**‡**^ **[p-value]**							
Medication			1.3 (0.7, 2.2) [0.404]	1.1 (0.6, 2.0) [0.676]	1.9 (0.9, 3.9) [0.068]	0.8 (0.4, 1.5) [0.415]	1.2 (0.7, 2.2) [0.463]
Assistive devices			1.1 (0.6, 2.4) [0.730]	0.8 (0.4, 1.8) [0.618]	1.9 (0.7, 5.2) [0.190]	0.6 (0.3, 1.5) [0.289]	0.7 (0.3, 1.5) [0.344]
Rehabilitation therapy			0.2 (0.1, 0.8) [0.016]	0.2 (0.1, 0.5) [0.001]	0.3 (0.1, 0.9) [0.040]	0.2 (0.1, 0.4) [<0.001]	0.3 (0.1, 0.8) [0.015]
Home care services			2.5 (0.9, 6.4) [0.063]	1.0 (0.4, 2.5) [0.964]	2.0 (0.6, 6.8) [0.257]	1.0 (0.4, 2.3) [0.962]	2.2 (1.0, 5.1) [0.053]

^†^Weighted percentages

^‡^Odds ratios were adjusted for age, sex and income

^§^Reference category

## Discussion

Our paper examined support service utilization and out-of-pocket health-care payments of people with neurological conditions from a nationally representative survey. While the study was undertaken in Canada, many regions in the world (e.g., North America, Europe, Australia) have similar models of assistance for people with chronic neurological conditions with very limited coverage by government services or health insurance. Like Canada, many countries (e.g. France, Japan, Australia, Norway, Italy) have national strategies for quality care of chronic conditions, including the delivery of support services [15]. Overall, our results demonstrate that Canadians with neurological conditions receive more informal than formal assistance. This observation has major implications on the potential health of caregivers. A 2015 study found that greater informal hours was a risk factor for caregiver distress when caring for people with neurological conditions; conversely, the provision of formal care-giving had a protective effect on caregiver distress [16].

We also observed that lower income was associated with less formal assistance, but not informal instance with care. This suggests that those in the lower income brackets rely less on formal assistance for their care. In addition, our study found that lower income was associated with having more rehabilitation therapy expenses. Prompt access to rehabilitation programs and services may decrease complication rates and improve functional outcomes. As government-sponsored health-care services (including rehabilitation) continue to shrink,[17] those with lower tax income may find it difficult to access the necessary rehabilitation services to optimize their recovery. Sixty-five percent of Canadians have private health insurance which pays for more than 90% of the approximately 11% of total healthcare expenditures that go towards other professionals, including rehabilitative care [18]. While the presence of private insurance has been associated with better outcomes after stroke [19], health insurance is mostly offered through employers and many stroke survivors will be retired or unable to return to work after stroke [20]. Thus, the negative economic impact after stroke can pose barriers to access to healthcare services.

Our results showed that individuals with stroke reported the lowest proportion of formal support service use, and second lowest proportion of informal support service use, but with generally lower odds of receiving any informal support services relative to the other non-stroke neurological conditions. This implies that individuals living with a stroke receive less support services than individuals with other adult neurological conditions. In addition to limitations in physical functioning, stroke often causes difficulties with awareness, attention, learning, memory, and communication [21]. This raises the question whether the stroke group did not truly require any assistance, or alternatively, could not advocate for the essential support services and hence were not receiving support as much as the other groups. All 6 conditions had at least three-quarters of their sample report restrictions in activities. Thus, despite having restrictions in activities, it is likely that individuals with stroke might not have had sufficient access to support services because they did not recognize or were unable to communicate their need for such services. We did run a post-hoc analysis of logistic regression models limited to individuals with restriction in activities in the sample, and the results were similar to the models with the sample population, which also included a small proportion without restriction in activities. Exploring barriers and facilitators to accessing support services by stroke survivors and adults living with other neurological conditions would be an important next step.

Generally, samples with conditions not caused by trauma (SCI, TBI) were far more likely to manage their care informally. Such a finding might be partly explained by financial compensation, which often accompanies traumatic injuries and enables formal care support. The chance of the ADD group receiving informal assistance with medical care, managing care and transportation was 7 to18 times larger compared to the stroke group and may suggest that mental abilities are critical for these functions; ADD has the highest prevalence of impaired cognition (93.7%) among neurological conditions in Canada [1]. While stroke doubles an individual’s risk for dementia (including Alzheimer’s disease) [22], the overall impact on cognition is likely less than in ADD. Various visual, motor and cognitive impairments common in neurological conditions affect complex tasks such as the ability to drive. Approximately one-third of survivors do return to driving after stroke [23]. Meanwhile, driving skills worsen as Alzheimer's disease progresses [24], eventually leading to driving cessation. This may be one reason for the higher odds of the ADD sample receiving informal assistance for transportation compared to stroke. Unlike older drivers with other disabilities who may resort to other modes of transport such as public transport, many individuals with ADD are unable to adapt and experience a lack of orientation in public transport stations [25].

Stroke survivors were more likely to make out-of-pocket payments for rehabilitation probably because many of the requisite services are not covered by the health-care system, and because rehabilitation services are delivered over a short window after stroke, while recovery can take months to occur. Prompt access to rehabilitation programs and services may decrease complication rates and improve functional outcomes. As government-sponsored health-care services (including rehabilitation) continue to shrink [17], those with lower taxable income may find it difficult to access the necessary rehabilitation services to optimize their recovery. As acute and rehabilitation stays continue to shorten, there has been an increase in early supported discharge in the community [26]. Home rehabilitation services cost less than hospital-based rehabilitation [27]. However, patients are often sent home even if there are not sufficient services in the community to care for them [28]. This predisposes them to the use of informal home care services for necessary activities. Many Canadians in long-term care facilities have ADD (43%), compared to those with stroke (15.4%), PD (5.1%), TBI (2.5%), MS (1.4%) and SCI (0.5%) [1]. This implies that a higher proportion of individuals with stroke live in the community than those with ADD.

There are other factors that may influence the use of support services in individuals with neurological conditions. For example, patient clinical characteristics (such as low cognition, poor activities of daily living performance), higher informal care hours and co-residence of the patient and caregiver have been associated with caregiver stress among individuals living in the community with neurological conditions [16]. Social support can help to deal with the consequences of neurological conditions and promote functional independence and quality of life [29–31]. Further research is needed to determine the influence of characteristics such as severity, duration and living arrangement on the use of support services.

There are limitations that warrant acknowledgment in our study. Firstly, all data were self-reported by individuals and may be biased by social desirability and recall errors. The study involved people living in private households therefore, the results may not be generalized to all stroke survivors as there are some who are residents of health-care institutions. The survey determined whether individuals received formal and informal support (i.e., presence/absence), but not the amount of support, which may answer our questions with greater clarity. The survey did not include non-neurological conditions (e.g., arthritis, heart disease) that may affect the use of support services, which could further bias our findings. The distribution of the conditions in our study is relatively similar to statistics in Canada [32], except those of TBI and ADD. They were less for TBI, possibly because we only included those with symptoms more than 6 months for TBI. They were less for ADD, possibly because individuals could not participate due to low cognition and also because we excluded those with multiple neurological conditions and the majority of those excluded had multiple conditions with ADD.

## Conclusions

Our findings suggest that Canadians with neurological conditions receive more informal assistance than formal assistance. Furthermore, it appears that stroke survivors receive less support services, while those with Alzheimer’s disease/dementia receive the most compared to other adult neurological conditions. Such data can help inform the development of support services in the community.

## Supporting information

S1 Table Logistic regression for formal assistance use and socio-demographic covariates (age, sex and income). (DOCX)

S2 Table Logistic regression for informal assistance use and socio-demographic covariates (age, sex and income). (DOCX)

S3 Table Logistic regression for out-of-pocket payments and socio-demographic covariates (age, sex and income). (DOCX)
