# Supplementary material for: Support service utilization and out-of-pocket payments for health services in a population-based sample of adults with neurological conditions
Source: PLoS One. 2018 Feb 23;13(2):e0192911. doi: 10.1371/journal.pone.0192911 (PMC5825050; doi:10.1371/journal.pone.0192911)
Supplement: S1 Table — (DOCX) [file pone.0192911.s001.docx]

**SUPPORTING INFORMATION**

**Support service utilization and out-of-pocket payments for health services in a population-based sample of adults with neurological conditions**

Adebimpe O. Obembe, PhD ^1,2^ , Charlie H. Goldsmith, PhD ^4,5,^, Lisa A. Simpson, MSc ^2,3^, Brodie M. Sakakibara, PhD ^1,2^, Janice J. Eng, PhD *^1,2^,

^1^Department of Physical Therapy, University of British Columbia, Vancouver, Canada

^2^Rehabilitation Research Program, GF Strong Rehab Centre, Vancouver Coastal Health Research Institute, Vancouver, Canada

^3^Graduate Program in Rehabilitation Sciences, University of British Columbia, Vancouver, Canada

^4^Adjunct Professor, Faculty of Health Sciences, Simon Fraser University, Burnaby, Canada
^5^Adjunct Professor, Department of Occupational Science and Occupational Therapy, Faculty of Medicine, The University of British Columbia, Vancouver, Canada

**S1 Table. Logistic regression for formal assistance use and socio-demographic covariates (age, sex and income)**

| **OR (95% CI)** | **Stroke**  (N=176452) | **Parkinson's disease**  (N=40753) | **Traumatic Brain Injury**  (N=61929) | **Spinal Cord Injury**  (N=50967) | **Multiple sclerosis**  (N=83957) | **Alzheimer's and dementias**  (N=45712) |
| --- | --- | --- | --- | --- | --- | --- |
| ***Personal care*** |  |  |  |  |  |  |
| Age | 3.09(1.69, 5.66)^*^ | 2.52(1.44, 4.42)^*^ | 2.36(1.31, 4.26)^*^ | 2.71(1.55, 4.72)^*^ | 2.67(1.51, 4.72)^*^ | 2.24(1.27, 3.97)^*^ |
| Sex | 1.61(1.00, 2.00)^†^ | 1.72(1.06, 2.78) ^†^ | 1.62(1.00, 2.61) ^†^ | 1.61(0.99, 2.63) | 1.63(1.02, 2.63) ^†^ | 1.57(0.97, 2.54) |
| Income | 0.53(0.34, 0.82) ^‡^ | 0.53(0.33, 0.84) ^‡^ | 0.53(0.33, 0.85) ^‡^ | 0.55(0.35, 0.87) ^‡^ | 0.55(0.35, 0.87) ^‡^ | 0.52(0.33, 0.81) ^‡^ |
| ***Medical care*** |  |  |  |  |  |  |
| Age | 2.94(1.66, 5.19)^*^ | 2.34(1.29, 4.25)^*^ | 2.16(1.25, 3.73)^*^ | 2.54(1.43, 4.50)^*^ | 2.46(1.35, 4.48)^*^ | 1.89(1.05, 3.41)^*^ |
| Sex | 1.26(0.78, 2.04) | 1.29(0.79, 2.11) | 1.26(0.77, 2.06) | 1.30(0.78, 2.16) | 1.25(0.75, 2.08) | 1.21(0.74, 2.00) |
| Income | 0.54(0.32, 0.93) ^‡^ | 0.57(0.33, 0.97) ^‡^ | 0.56(0.32, 0.99) ^‡^ | 0.57(0.33, 0.98) ^‡^ | 0.57(0.33, 0.99) ^‡^ | 0.54(0.31, 0.94) ^‡^ |
| ***Managing care*** |  |  |  |  |  |  |
| Age | 0.53(0.21, 1.34) | 0.35(0.12, 1.00) ^§^ | 0.68(0.30, 1.54) | 0.36(0.13, 1.04) | 0.34(0.11, 1.07) | 0.33(0.11, 1.04) |
| Sex | 2.32(0.84, 6.46) | 2.54(0.90, 7.22) | 2.56(0.90, 7.25) | 2.24(0.77, 6.49) | 2.63(0.86, 8.03) | 2.35(0.81, 6.83) |
| Income | 0.44(0.16, 1.27) | 0.44(0.15, 1.30) | 0.53(0.19, 1.44) | 0.45(0.15, 1.38) | 0.46(0.16, 1.35) | 0.44(0.15, 1.33) |
| ***Transportation*** |  |  |  |  |  |  |
| Age | 0.90(0.46, 1.74) | 0.79(0.36, 1.74) | 0.97(0.54, 1.75) | 0.80(0.36, 1.75) | 0.75(0.32, 1.73) | 0.74(0.33, 1.68) |
| Sex | 1.38(0.63, 3.02) | 1.38(0.61, 3.11) | 1.41(0.64, 3.09) | 1.39(0.63, 3.09) | 1.44(0.62, 3.34) | 1.37(0.61, 3.07) |
| Income | 0.57(0.25, 1.28) | 0.58(0.25, 1.33) | 0.61(0.28, 1.31) | 0.58(0.26, 1.32) | 0.58(0.26, 1.31) | 0.57(0.25, 1.33) |
| ***Emotional support*** |  |  |  |  |  |  |
| Age | 1.05(0.50, 2.22) | 0.88(0.36, 2.17) | 1.21(0.62, 2.38) | 0.88(0.38, 2.05) | 0.84(0.33, 2.14) | 0.81(0.33, 2.03) |
| Sex | 1.42(0.64, 3.12) | 1.46(0.63, 3.34) | 1.46(0.65, 3.27) | 1.37(0.61, 3.09) | 1.51(0.63, 3.60) | 1.39(0.61, 3.17) |
| Income | 0.67(0.28, 1.59) | 0.68(0.28, 1.64) | 0.73(0.33, 1.63) | 0.69(0.29, 1.67) | 0.69(0.29, 1.63) | 0.67(0.28, 1.64) |
| ***Household activities*** |  |  |  |  |  |  |
| Age | 1.72(1.11, 2.68)^*^ | 1.44(0.90, 2.30) | 1.63(1.04, 2.54)^*^ | 1.56(0.98, 2.48) | 1.51(0.93, 2.43) | 1.44(0.89, 2.33) |
| Sex | 1.50(0.90, 1.10) | 1.52(0.90, 2.60) | 1.50(0.90, 2.51) | 1.54(0.92, 2.60) | 1.48(0.88, 2.49) | 1.48(0.88, 2.50) |
| Income | 0.58(0.33, 1.03) | 0.71(0.44, 1.14) | 0.73(0.45, 1.20) | 0.71(0.44, 1.15) | 0.72(0.45, 1.15) | 0.71(0.44, 1.15) |

^*^Significantly associated with higher odds for people younger than 60 years

^†^Significantly associated with higher odds for women

^‡^Significantly associated with lower odds for people with lower income

^§^Significantly associated with lower odds for people younger than 60 years
