# Supplementary material for: Support service utilization and out-of-pocket payments for health services in a population-based sample of adults with neurological conditions
Source: PLoS One. 2018 Feb 23;13(2):e0192911. doi: 10.1371/journal.pone.0192911 (PMC5825050; doi:10.1371/journal.pone.0192911)
Supplement: S2 Table — (DOCX) [file pone.0192911.s002.docx]

**SUPPORTING INFORMATION**

**Support service utilization and out-of-pocket payments for health services in a population-based sample of adults with neurological conditions**

Adebimpe O. Obembe, PhD ^1,2^ , Charlie H. Goldsmith, PhD ^4,5,^, Lisa A. Simpson, MSc ^2,3^, Brodie M. Sakakibara, PhD ^1,2^, Janice J. Eng, PhD *^1,2^,

^1^Department of Physical Therapy, University of British Columbia, Vancouver, Canada

^2^Rehabilitation Research Program, GF Strong Rehab Centre, Vancouver Coastal Health Research Institute, Vancouver, Canada

^3^Graduate Program in Rehabilitation Sciences, University of British Columbia, Vancouver, Canada

^4^Adjunct Professor, Faculty of Health Sciences, Simon Fraser University, Burnaby, Canada
^5^Adjunct Professor, Department of Occupational Science and Occupational Therapy, Faculty of Medicine, The University of British Columbia, Vancouver, Canada

**S2 Table. Logistic regression for informal assistance use and socio-demographic covariates (age, sex and income)**

| **OR (95% CI)** | **Stroke**  (N=176452) | **Parkinson's disease**  (N=40753) | **Traumatic Brain Injury**  (N=61929) | **Spinal Cord Injury**  (N=50967) | **Multiple sclerosis**  (N=83957) | **Alzheimer's and dementias**  (N=45712) |
| --- | --- | --- | --- | --- | --- | --- |
| ***Personal care*** |  |  |  |  |  |  |
| Age | 1.78(1.13, 2.83)^*^ | 1.59(1.03, 2.47)^*^ | 1.42(0.91, 2.21) | 1.74(1.12, 2.72)^*^ | 1.58(1.02, 2.44)^*^ | 1.36(0.88, 2.12) |
| Sex | 0.94(0.63, 1.39) | 0.95(0.64, 1.42) | 0.92(0.62, 1.36) | 0.97(0.66, 1.44) | 0.96(0.65, 1.42) | 0.90(0.60, 1.33) |
| Income | 1.11(0.75, 1.64) | 1.12(0.76, 1.67) | 1.11(0.74, 1.65) | 1.12(0.76, 1.67) | 1.14(0.77, 1.69) | 1.10(0.74, 1.64) |
| ***Medical care*** |  |  |  |  |  |  |
| Age | 3.54(2.14, 5.87)^*^ | 3.20(2.01, 5.09) ^*^ | 2.99(1.87, 4.76)^*^ | 2.94(1.86, 4.65)^*^ | 2.76(1.71, 4.47)^*^ | 1.95(1.20, 3.15)^*^ |
| Sex | 0.87(0.58, 1.29) | 0.83(0.56, 1.24) | 0.86(0.58, 1.28) | 0.84(0.56, 1.25) | 0.92(0.61, 1.37) | 0.76(0.50, 1.15) |
| Income | 0.96(0.67, 1.38) | 1.03(0.71, 1.89) | 1.00(0.69, 1.46) | 1.01(0.70, 1.46) | 1.01(0.70, 1.46) | 0.91(0.62, 1.34) |
| ***Managing care*** |  |  |  |  |  |  |
| Age | 2.37(1.53, 3.69)^*^ | 2.02(1.29, 3.16)^*^ | 2.26(1.50, 3.42 ^*^ | 1.96(1.27, 3.05)^*^ | 1.69(1.06, 2.68)^*^ | 1.31(0.83, 2.07) |
| Sex | 0.96(0.64, 1.44) | 0.94(0.62, 1.43) | 0.96(0.64, 1.45) | 0.94(0.63, 1.41) | 1.07(0.70, 1.62) | 0.86(0.56, 1.33) |
| Income | 0.79(0.54, 1.16) | 0.84(0.57, 1.23) | 0.85(0.59, 1.23) | 0.84(0.58, 1.22) | 0.84(0.58, 1.23) | 0.75(0.51, 1.12) |
| ***Transportation*** |  |  |  |  |  |  |
| Age | 1.68(1.13, 2.50)^*^ | 1.41(0.95, 2.10) | 1.47(1.00, 2.16) | 1.49(1.01, 2.20)^*^ | 1.28(0.86, 1.93) | 1.07(0.70, 1.62) |
| Sex | 2.46(1.67, 3.63)^†^ | 2.43(1.66, 3.56)^†^ | 2.42(1.65, 3.55)^†^ | 2.50(1.72, 3.64)^†^ | 2.60(1.77, 3.81)^†^ | 2.38(1.60, 3.55) ^†^ |
| Income | 0.80(0.55, 1.16) | 0.83(0.58, 1.19) | 0.84(0.59, 1.19) | 0.83(0.58, 1.19) | 0.84(0.59, 1.20) | 0.79(0.54, 1.16) |
| ***Emotional support*** |  |  |  |  |  |  |
| Age | 1.50(1.00, 2.24)^*^ | 1.33(0.89, 1.99) | 1.31(0.89, 1.94) | 1.39(0.95, 2.06) | 1.26(0.83, 1.90) | 1.07(0.70, 1.64) |
| Sex | 1.73(1.18, 2.55)^†^ | 1.73(1.18, 2.53)^†^ | 1.71(1.17, 2.51)^†^ | 1.76(1.20, 2.57)^†^ | 1.80(1.22, 2.66)^†^ | 1.67(1.13, 2.47) ^†^ |
| Income | 0.94(0.66, 1.34) | 0.96(0.68, 1.35) | 0.96(0.68, 1.34) | 0.96(0.68, 1.35) | 0.97(0.69, 1.36) | 0.93(0.64, 1.33) |
| ***Household activities*** |  |  |  |  |  |  |
| Age | 1.28(0.88, 1.85) | 1.08(0.74, 1.56) | 1.06(0.73, 1.53) | 1.17(0.81, 1.68) | 1.02(0.69, 1.52) | 0.85(0.58, 1.25) |
| Sex | 1.34(0.95, 1.88) | 1.32(0.94, 1.87) | 1.32(0.93, 1.86) | 1.40(1.00, 1.97)^†^ | 1.37(0.96, 1.95) | 1.27(0.90, 1.79) |
| Income | 0.79(0.56, 1.11) | 0.83(0.59, 1.16) | 0.82(0.59, 1.16) | 0.81(0.58, 1.14) | 0.78(0.50, 1.22) | 0.79(0.56, 1.11) |

^*^Significantly associated with higher odds for people younger than 60 years

^†^Significantly associated with higher odds for women
