# Supplementary material for: Support service utilization and out-of-pocket payments for health services in a population-based sample of adults with neurological conditions
Source: PLoS One. 2018 Feb 23;13(2):e0192911. doi: 10.1371/journal.pone.0192911 (PMC5825050; doi:10.1371/journal.pone.0192911)
Supplement: S3 Table — (DOCX) [file pone.0192911.s003.docx]

**SUPPORTING INFORMATION**

**Support service utilization and out-of-pocket payments for health services in a population-based sample of adults with neurological conditions**

Adebimpe O. Obembe, PhD ^1,2^ , Charlie H. Goldsmith, PhD ^4,5,^, Lisa A. Simpson, MSc ^2,3^, Brodie M. Sakakibara, PhD ^1,2^, Janice J. Eng, PhD *^1,2^,

^1^Department of Physical Therapy, University of British Columbia, Vancouver, Canada

^2^Rehabilitation Research Program, GF Strong Rehab Centre, Vancouver Coastal Health Research Institute, Vancouver, Canada

^3^Graduate Program in Rehabilitation Sciences, University of British Columbia, Vancouver, Canada

^4^Adjunct Professor, Faculty of Health Sciences, Simon Fraser University, Burnaby, Canada
^5^Adjunct Professor, Department of Occupational Science and Occupational Therapy, Faculty of Medicine, The University of British Columbia, Vancouver, Canada

**S3 Table. Logistic regression for out-of-pocket payments and socio-demographic covariates (age, sex and income)**

| **OR (95% CI)** | **Stroke**  (N=176452) | **Parkinson's disease**  (N=40753) | **Traumatic Brain Injury**  (N=61929) | **Spinal Cord Injury**  (N=50967) | **Multiple sclerosis**  (N=83957) | **Alzheimer's and dementias**  (N=45712) |
| --- | --- | --- | --- | --- | --- | --- |
| ***Medication*** |  |  |  |  |  |  |
| Age | 1.21(0.83, 1.75) | 1.17(0.79, 1.74) | 1.04(0.71, 1.53) | 1.27(0.86, 1.87) | 1.18(0.79, 1.75) | 1.14(0.76, 1.71) |
| Sex | 1.39(0.98, 1.96) | 1.39(0.98, 1.97) | 1.37(0.97, 1.94) | 1.47(1.04, 2.09) ^†^ | 1.39(0.96, 1.99) | 1.38(0.97, 1.96) |
| Income | 1.07(0.77, 1.49) | 1.07(0.77, 1.49) | 1.05(0.76, 1.45) | 1.06(0.76, 1.18) | 1.07(0.77, 1.49) | 1.07(0.77, 1.48) |
| ***Assistive devices*** |  |  |  |  |  |  |
| Age | 1.52(1.01, 2.28)^*^ | 1.38(0.88, 2.16) | 1.21(0.77, 1.89) | 1.51(0.98, 2.31) | 1.58(1.02, 2.46)^*^ | 1.42(0.90, 2.23) |
| Sex | 1.15(0.77, 1.71) | 1.16(0.77, 1.75) | 1.12(0.75, 1.68) | 1.20(0.80, 1.80) | 1.06(0.70, 1.63) | 1.14(0.77, 1.70) |
| Income | 0.95(0.65, 1.40) | 0.96(0.65, 1.42) | 0.94(0.64, 1.38) | 0.96(0.65, 1.41) | 0.96(0.66, 1.41) | 097(0.66, 1.43) |
| ***Rehabilitation Therapy*** |  |  |  |  |  |  |
| Age | 0.80(0.47, 1.35) | 0.79(0.42, 1.51) | 0.79(0.44, 1.44) | 0.90(0.51, 1.61) | 0.81(0.45, 1.46) | 1.01(0.54, 1.88) |
| Sex | 1.64(0.99, 2.73) | 1.72(1.04, 2.86)^†^ | 1.63(0.98, 2.73) | 1.77(1.04, 3.00) ^†^ | 1.69(0.97, 2.96) | 1.73(1.03, 2.93) ^†^ |
| Income | 2.00(1.06, 3.78)^‡^ | 1.94(1.05, 3.58)^‡^ | 1.95(1.07, 3.55) ^‡^ | 1.97(1.07, 3.60) ^‡^ | 1.98(1.08, 3.63) ^‡^ | 2.07(1.11, 3.83) ^‡^ |
| ***Household services*** |  |  |  |  |  |  |
| Age | 2.16(1.33, 3.52)^*^ | 1.70(0.96, 3.02) | 1.77(1.07, 2.95)^*^ | 2.03(1.21, 3.38)^*^ | 1.72(0.98, 3.01) | 1.60(0.91, 2.81) |
| Sex | 1.21(0.69, 2.09) | 1.26(0.71, 2.25) | 1.19(0.68, 2.07) | 1.28(0.71, 2.31) | 1.24(0.70, 2.20) | 1.15(0.66, 2.01) |
| Income | 1.30(0.76, 2.21) | 1.32(0.77, 3.71) | 1.35(0.79, 2.32) | 1.34(0.80, 2.26) | 1.37(0.81, 2.31) | 1.33(0.78, 2.25) |

^*^Significantly associated with higher odds for people younger than 60 years

^†^Significantly associated with higher odds for women

^‡^Significantly associated with higher odds for people with lower income
